# On the way to the 10 ps time-of-flight PET challenge

**DOI:** 10.1140/epjp/s13360-022-03159-8

**Published:** 2022-08-26

**Authors:** P. Lecoq

**Affiliations:** 1 Instituto de Instrumentación Para Imagen Molecular (I3M), Valencia, Spain; 2 Multiwave Metacrystal S.A., Geneva, Switzerland; 3 CERN, Geneva, Switzerland

## Abstract

There is a consensus for gathering the multidisciplinary academic and industrial medical imaging community around the ambitious challenge to develop a 10 ps Time-of-Flight PET scanner (TOFPET). The goal is to reduce the radiation dose (currently 5–25 mSv for whole-body PET/CT) and/or scan time (currently > 10 min) by an order of magnitude, with a significant gain in the patient comfort and cost per exam (currently in the range of 1000 € per scan). To achieve this very ambitious goal it is essential to significantly improve the performance of each component of the detection chain: light production, light transport, photodetection, readout electronics. Speeding up progress in this direction is the goal of the challenge and will have an important impact on the development of a new generation of ionization radiation detectors. The possibility to reach 10 ps time-of-flight resolution at small energies (511 keV), as required in finely granulated calorimeters and PET scanners, although extremely challenging, is not limited by physical barriers and a number of disruptive technologies, such as multifunctional heterostructures, combining the high stopping power of well-known scintillators with the ultrafast photon emission resulting from the 1D, 2D or 3D quantum confinement of the excitons in nanocrystals, photonic crystals, photonic fibers, as well as new concepts of 3D digital SiPM structures, open the way to new radiation detector concepts with unprecedented performance.

## Introduction

The challenge for functional isotopic imaging lays in its capacity to quantitatively measure the relative metabolic activity of the specific molecular pathways in action in a metabolic process. To achieve this, it is necessary to improve both the imaging system’s spatial resolution, that is, its capacity to discriminate two separate objects, and the PET sensitivity, i.e. it’s capacity to detect very small amounts of radiotracer uptake in the smallest possible image voxels. An important point is to achieve a good image contrast to noise ratio (CNR) in order to precisely evaluate a metabolic agent’s concentration in an organ or group of cells. The precision of the concentration's measurement depends mainly, but not only, on the imaging system’s sensitivity, and therefore its capacity to accumulate the statistics needed to reconstruct in vivo the 4D (space and time) distribution of the radiopharmaceutical. The CNR is related to the variance of the signal in each image voxel, which is the square of the signal-to-noise ratio (SNR).

A significant improvement in the SNR can be obtained by pushing the limits of Time-Of-Flight PET techniques (TOFPET), resulting in a corresponding clinical sensitivity increase and dose reduction potential according to Eq. (1):

1 $$ {\raise0.7ex\hbox{${{\text{SNR}}_{{{\text{TOF}}}} }$} \!\mathord{\left/ {\vphantom {{{\text{SNR}}_{{{\text{TOF}}}} } {{\text{SNR}}_{{{\text{NONTOF}}}} }}}\right.\kern-\nulldelimiterspace} \!\lower0.7ex\hbox{${{\text{SNR}}_{{{\text{NONTOF}}}} }$}} = \sqrt {\frac{2D}{{c.{\text{CTR}}}}} $$

where *D* is the diameter of the object, *c* is the speed of light in vacuum, *CTR* is the Coincidence Time Resolution.

The best scanner today with respect to TOF (Siemens Biograph Vision) has a resolution of 214 ps [1]. However, most current PET scanners have a resolution of 400–600 ps. The recently launched 10 ps Time-of-Flight PET challenge [2] is proposing a target resolution of 10 ps.

Pushing the research in this direction is of paramount importance in the context of personalised medicine, and particularly for the hot topic of immunotherapy (2018 Nobel Prize in Physiology or Medicine) for the challenging tracking of immune cells and cancer stem cells. Other consequences of introducing sub-100 ps TOFPET are:

- Reduction of radiation doses to negligibly low levels, allowing more and better longitudinal studies in human patients,
- Reduction in the needed quantity and costs of radiopharmaceutical for in vivo molecular imaging procedures,
- Extension of the benefit of molecular imaging procedures beyond oncology towards cardiovascular, neurological, metabolic, inflammatory, infectious or metabolic diseases, including at the pediatric, neonatal, and prenatal levels,
- suppression of artifacts in PET system with limited angle tomography for guiding biopsy or in operation rooms, allowing also partial ring lower cost scanners,
- for precise dynamic studies of molecular processes of high interest in pharmacology to screen and select new drugs.

Moreover, reaching 10 ps CTR resolution will allow a detailed recording of the whole Compton-photoelectric interaction chain in the crystals and the recovery of part of the scattered events with a strong impact on both the spatial resolution and on the sensitivity of the imaging devices.

Although extremely challenging, it has been shown that this objective is not limited by physical show-stoppers and that a number of disruptive technologies, applicable to all the components of the detection chain (light production, light transport, light conversion, electronic readout, image reconstruction) open the way to its realization [3]. Several groups worldwide are contributing to this ambitious goal and their visions are summarized in an interesting roadmap [4] with a number of references on all these subjects herein.

## Light production: metascintillators

The dynamics of the scintillation light produced by an inorganic scintillator results from a complex sequence of relaxation mechanisms of the hot electron–hole pairs produced by the interaction of ionizing radiation with the scintillator crystal before the luminescent centers of the scintillator can be activated. This relaxation process is at the origin of the scintillation rise time *τ*_r_, which delays the emission of the first produced photons, increases their time jitter and reduces accordingly the time resolution of the scintillator. The process being stochastic large statistical fluctuations are therefore induced for the generation of the first scintillation photons, which sets an intrinsic limit to the time resolution that can be achieved by a scintillator. The scintillator light yield LY and the scintillation decay time *τ*_d_, determined by the oscillator strength of the optical transition between the luminescent excited and fundamental states, play also an important role in the coincidence time resolution that can be achieved with a scintillator, according to the following formula:

2 $$ {\text{CTR}} \propto \sqrt {\frac{{\tau_{r} \tau_{d} }}{LY}} $$

Considering that *L*(*Y*)SO, the most popular crystal used in PET scanners, with a *LY* of 40.000 ph/MeV, a decay time of 40 ns and a rise time of 70 ps cannot produce more than 0.5 photons/ps at 511 keV, and that this number is further reduced by absorption and photoconversion losses, it is unlikely that a CTR of 100 ps or better can be reached with standard scintillator technology. The best inorganic scintillators emitting light in the visible range are not far from reaching the limits for the light yield and the decay time. This is related to energy conservation laws in the transition matrix strength of the allowed transitions and improvements over a factor of 10, necessary for 10 ps TOF-PET, are virtually impossible [5].

However, it has been shown [3] that the addition of a moderate number of prompt photons to the scintillation light could significantly improve the timing performance. Different sources of prompt photons are presently investigated, such as Cerenkov emission, hot intraband luminescence, organic scintillation mechanisms, nanocrystals. Nanoscintillators are particularly interesting, as the quantum confinement of excitons in nanocrystals leads to ultrafast sub-ns emission with a quantum efficiency approaching unity [4].

**Fig. 1:**
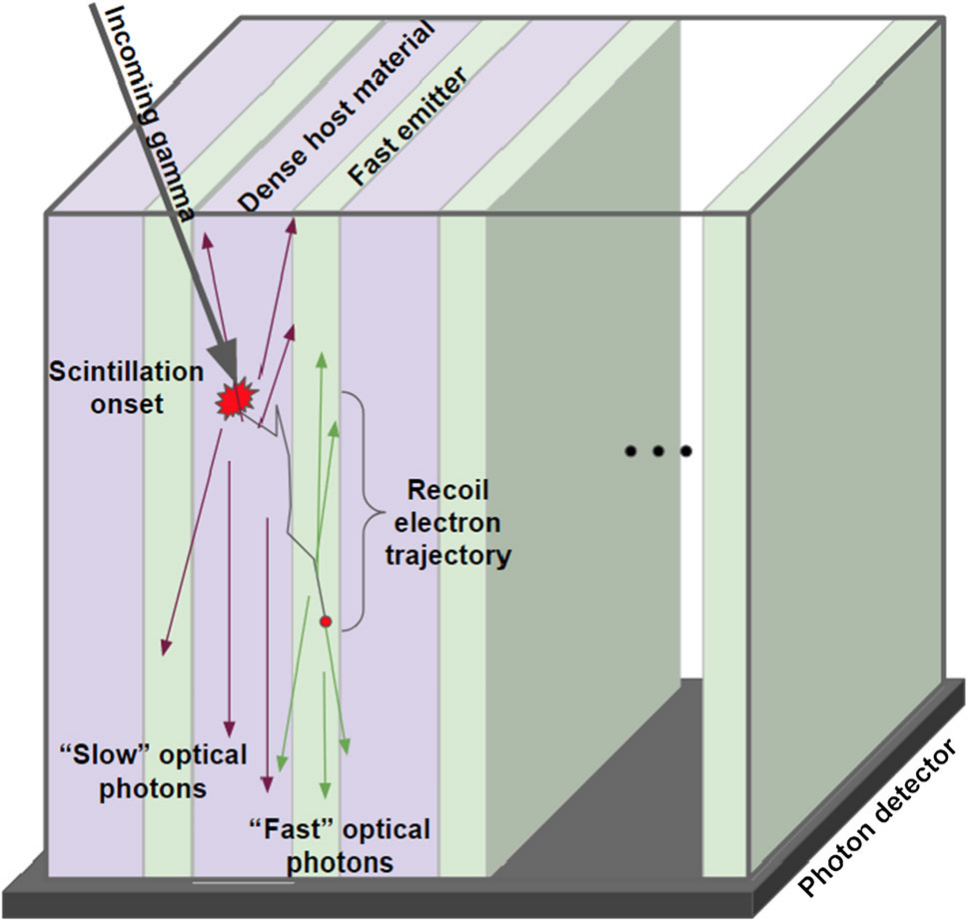
Example of a metascintillator made of alternate layers of thin slices of a dense host material and a fast emitter. (From ref [6])

This is leading to the concept of metacrystal [6, 7], an heterostructure combining the high stopping power and good energy resolution of standard scintillators (L(Y)SO, BGO, NaI, CsI, GAGG, etc.…) with a source of prompt photons. The recoil electron produced by the conversion of the incident *γ*-ray in the dense scintillator is sampled in a thin layer of nanocrystals, producing a bunch of prompt photons, allowing a precise time tag of the event, as shown in Fig. 1.

Following very encouraging results already reported in [6, 7] different metascintillator topologies, with several types of fast emitters are presently being investigated. Progress in cheap production of ultrafast scintillators, material nanostructuration, scintillator 3D printing, etc.…, offers interesting perspectives to design complex and efficient scintillator heterostructures. A first objective is to reach 100 ps CTR resolution with a LYSO-based configuration. There is also an interest for reaching the same CTR of 200 ps as the present state-of-the art using bulk LYSO, but with a BGO-based configuration, BGO being about 3 times cheaper than LYSO.

## Light transport: photonic crystals

Inorganic scintillators are generally characterized by a high refractive index, as a consequence of the required high density to provide the necessary stopping power for ionizing radiation. The index mismatch between the crystal and the surrounding medium (air or optical grease) helps guiding the light in the very thin plates of the metascintillator through the total reflection (TIR) of the photons in a large solid angle. However, it strongly limits the light extraction efficiency and increases the travel path and the absorption probability through multiple bouncing of the photons in the crystal. Increasing the light extraction efficiency will improve the timing resolution through a better photo-statistics but also by reducing the number pf photons bouncing in the crystal before having a chance to be extracted. Indeed, in a real setup with 20 mm long crystal, each bouncing adds several hundreds of ps to the photon travel time.

Nanostructures, such as photonic crystals (PhCs), permit extraction of the scintillation light beyond the critical angle, thereby substantially improving the light extraction efficiency as well as energy and timing resolution [3, 8].

The use of photonic crystals, which are nano-structured media with a periodic modulation of the dielectric constant [9, 10] allows the evanescent wave, associated to photons reaching the crystal extraction face at large angles to constructively interfere and to produce real extraction modes. As compared to the conventional shallow gratings, the strong index variation induced by the photonic crystal gives rise to an extracting efficiency more tolerant to the wavelength and to the incidence angle. This can be exploited to couple light propagation modes inside and outside the crystal and to force some of the photons, normally trapped in the crystal by total internal reflection, to escape.

**Fig. 2:**
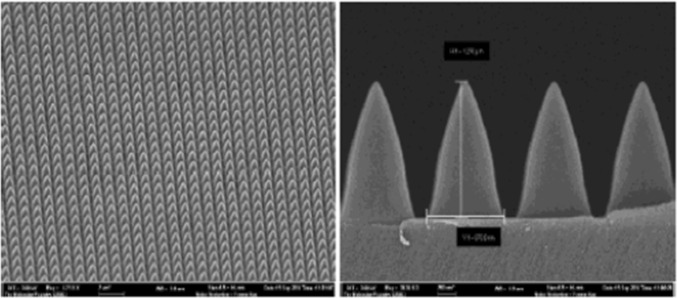
Scanning electron microscope (SEM) image of the silicon template used for nanoimprinting in the high index of refraction polymers. The nanocones have a base diameter of 880 nm, and a height of 1275 nm, as indicated by the horizontal and vertical scale bars, respectively. (from ref [11])

Simple, cost-effective methods have been developed to produce nanoimprinted PhCs on high index polymers [11] from a nano-structured Si stamp (Fig. 2).

**Fig. 3.**
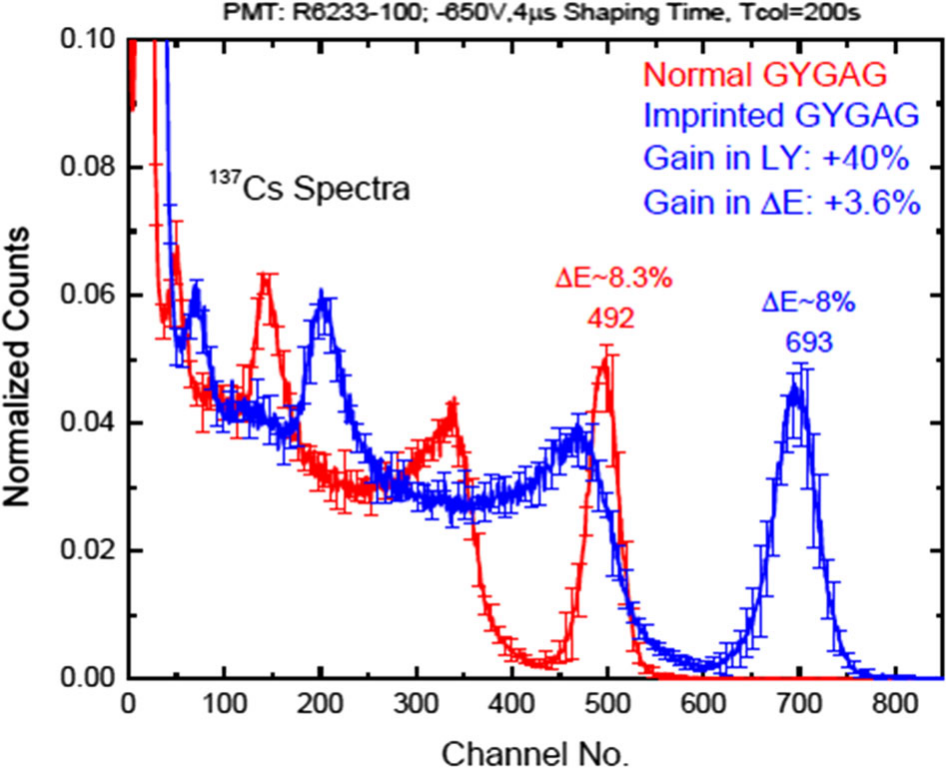
^137^Cs spectra of non-imprinted (red curve) and nanoimprinted (blue curve) GYGAG. Imprinting was carried out in a polymer having RI *n* = 1.825 @ 550 nm. Nanoimprinted GYGAG demonstrated a 40% enhancement in light yield, and 3.6% improvement in the energy resolution. (from ref [11])

Impressive light yield, and corresponding energy resolution gains have been obtained, as shown on Fig. 3.

**Fig. 4:**
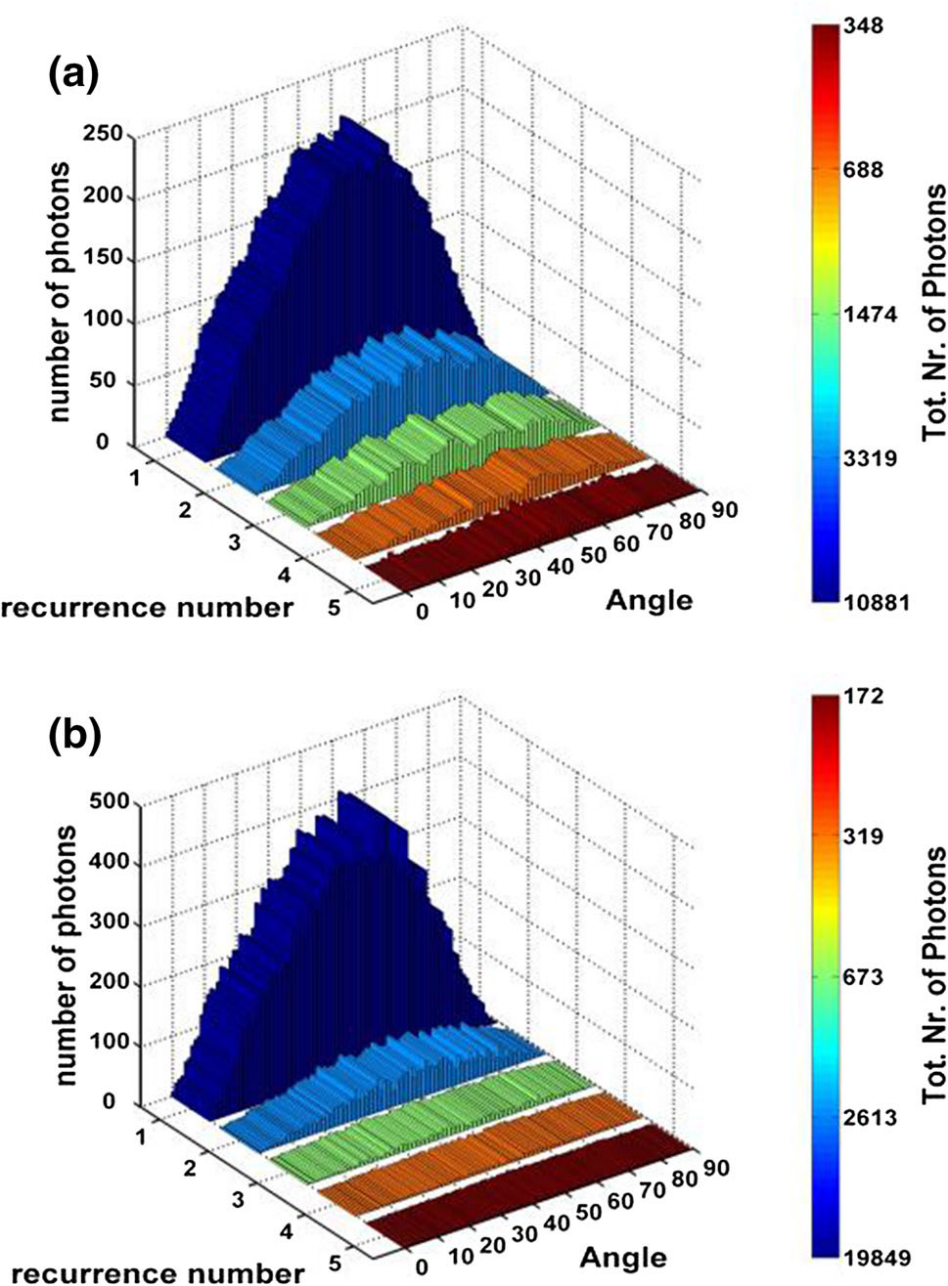
Angular distribution of photons at each recurrence on the extraction face, **a** without PhC, **b** with PhC The number of photons bouncing in the crystal is significantly reduced with the PhC. (from ref [12])

This LY increase leads of course to a gain in timing though improved photo-statistics, but also by the fact that a PhC grating modifies the weight of the different light propagation modes in the crystal [12]. More photons are being extracted the first time they hit the ‘out-coupling’ face of the crystal, increasing therefore the number of fast photons and decreasing the number of back reflected photons reappearing later, as shown in the light ray tracing simulation plots in Fig. 4.

## Light conversion

Improving the timing resolution of scintillator-based detectors requires also a large effort in the development of a new generation of photodetectors, with a low dark count rate (DCR) and improved single photon time resolution (SPTR) by an order of magnitude as compared to about 100 ps today. Photodetectors are developing along several axes, such as miniaturized vacuum tubes, multichannel plates (MCP), large area avalanche photodiodes (LAAPD), silicon photomultipliers (SiPM), involving new detecting materials, 3D IC technology, cryogenic operation, and deep learning.

In particular, the possibility of combining the extraordinary potential of nanophotonics with new approaches offered by modern microelectronics and 3D electronic integration opens novel perspectives for the development of a new generation of metamaterial-based SiPMs with unprecedented photodetection efficiency and timing resolution. This is the basis of the concept of Quantum Silicon Detector (QSD) described in [13].

SiPMs are composed of a large number of Single Avalanche Photodiodes (SPADs). They are separated by dead borders at the edges for electrical and optical isolation and to prevent edge breakdown. This reduces the so-called fill-factor of the device and sets an intrinsic limit to its photodetection efficiency (PDE).

Several microlens solutions have been investigated to focus the incoming light to the SPADs, and preferably to the central region of the SPADs, where the electric field does not suffer from edge effects (see for instance [14]). If perfectly applicable for collimated light, for instance for LIDAR applications, this approach is not efficient for scintillator applications, with a large angular distribution of the photons at the output face of the scintillator.

**Fig. 5:**
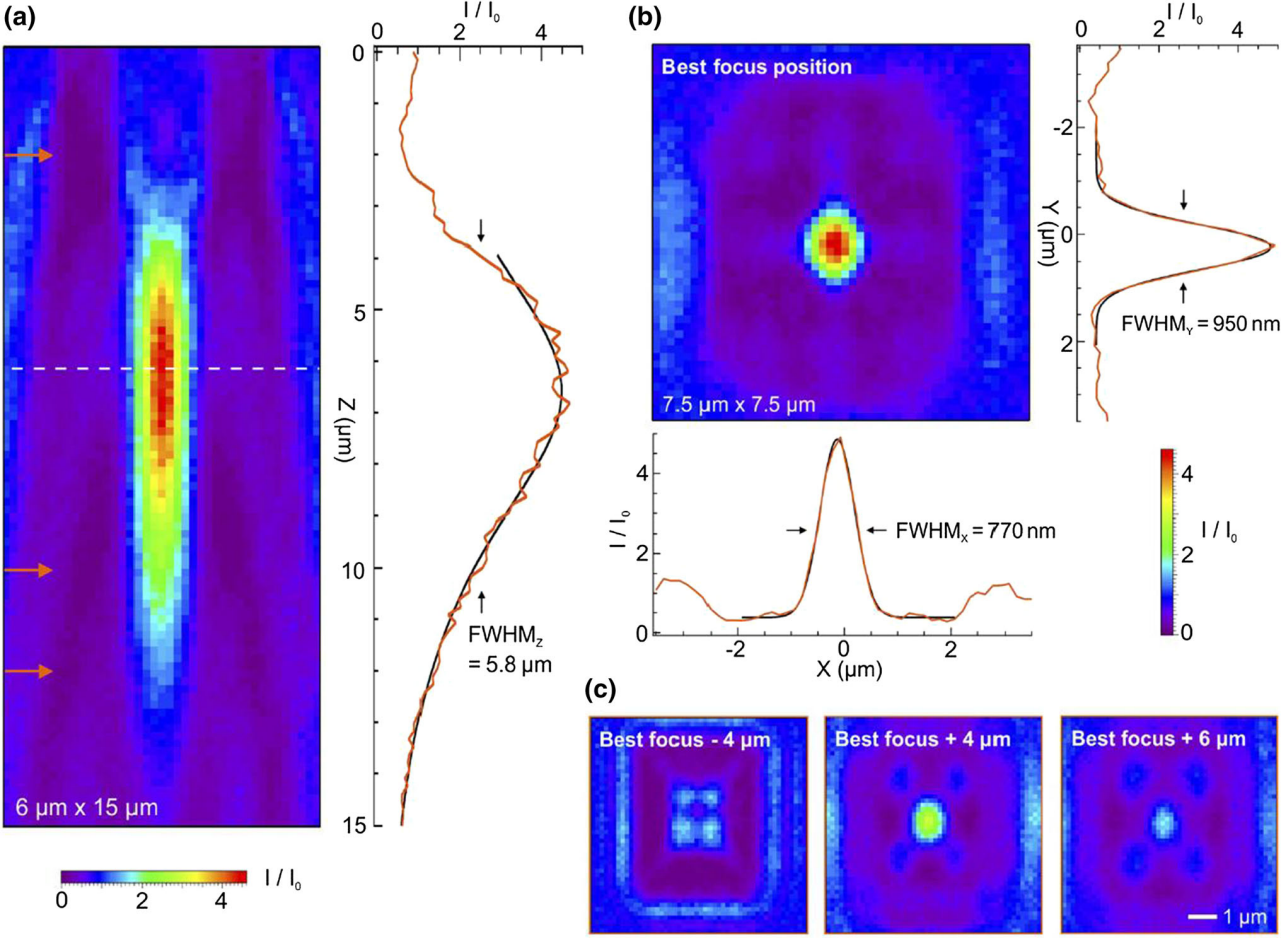
Experimental characterization of the metalens. **a** Scanning optical microscope image along the *XZ*-plane parallel to the light propagation. **b** Microscope image recorded at the best focus position [the white dashed line in (**a**)] along the *XY*-plane perpendicular to the beam propagation. **c** Images recorded in the transverse plane at different distances from the best focus position [defined in (**b**)], corresponding to the positions indicated by the orange arrows in (**a**). All the images share the same color scale. (from ref [15])

One solution to overcome the fill-factor limitations is to use transformation optics concentrators or hyperlenses with a high light concentration power over a large spectral range and angular acceptance, to guide photons to the SPAD. Such plasmonic solutions provide an elegant way to improve the fill-factor and hence the photodetection efficiency compared to conventional SiPMs and to boost the PDE to values approaching 100%. The potential of metasurfaces to be used as light concentrators has been studied for several years and has been demonstrated in many other applications. In this regard, achieving light concentration by means of resonant plasmonic structures constitutes a true paradigm shift in the field of single photon detection. Indeed, it is possible to focus light in a very small central spot of the microcell, obtaining almost 100% fill-factor for light detection while leaving plenty of space to increase the width of the dead border even in very small cells (≤ 5 μm), as demonstrated in [15], where 93% of the incident light is concentrated in a less than 1 μm spot size by a gradient index (GRIN) lens (Fig. 5).

Very small cell pitch of ≤ 10 μm is important to reduce as much as possible the gain, the primary and correlated noise, as well as the dead time and thus, preserve all the information carried by the light quanta. Such small cells will also increase the cell density and significantly extend the QSD linear dynamic range. Backside illumination allows enhancing the Fill Factor and move the Back End of Line (BEOL) to the opposite side with respect to the light entrance window, thus providing an optimal surface to apply nanophotonic enhancements.

Another source of time jitter in a SiPM is related to the statistical variation of the depth of interaction of the photon in the SiPM structure, as a function of its wavelength and incident angle. The use of hyberbolic metamaterials gives the possibility to create strong spot-like photoconversion regions in a well-defined position of the SPAD, making the detection process tunable and the electronic avalanche generation independent from the photon’s impinging point, wavelength and direction [16]. The integration of such hyperbolic metamaterial layers in the SiPM structure is expected to improve SPTR and PDE of the device.

Finally, highly integrated front-end electronics readout, including time digitizers for each individual SPAD or for a very small group of SPADs in a 3D electronics architecture layout, will allow picking-up the time information as close as possible to the point where it is generated, while reducing the difference in propagation delay, the noise and the capacitance of the photon detection chain. More details are given in reference [13].

**Fig. 6:**
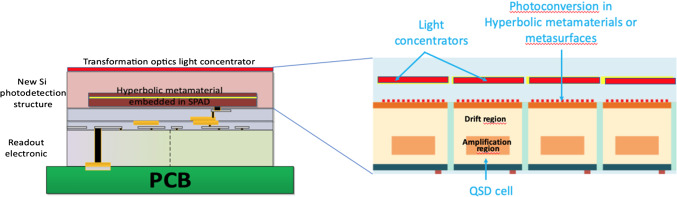
Principle of the Quantum Silicon Detector. (from ref [13])

A sketch of the concept of the new QSD photodetector is shown in Fig. 6.

## Conclusions

The quest for TOFPET of ever better timing performance is justified by new medical challenges requiring a very high sensitivity to open the way to dynamic parametric imaging, stem cell imaging, cancer immunotherapy follow-up, deciphering the complex functioning of our immune system and understanding how it is leading to the cytokine storm in the some covid-19 patients, etc.…

Reaching the ultimate goal of 10 ps CTR resolution, as encouraged by the 10 ps TOFPET challenge is obviously very ambitious. But a number of emerging technologies open the way to a paradigm shift in PET imaging. Some of them have been directly addressed in this paper, such as metascintillators, nanophotonics, microelectronics. But we should also mention scintillator growth technology, including 3D printing [17], as well as artificial intelligence for data analysis and image reconstruction [18]. The combination of all these technologies will allow directly recording the 3D information of the positron decay on an event-to-event basis with a millimetric resolution, and boosting the effective sensitivity by at least an order of magnitude as compared to the present state-of-the-art.

## Data Availability

No Data associated in the manuscript.
